# T Cells Detect Intracellular DNA but Fail to Induce Type I IFN Responses: Implications for Restriction of HIV Replication

**DOI:** 10.1371/journal.pone.0084513

**Published:** 2014-01-03

**Authors:** Randi K. Berg, Stine H. Rahbek, Emil Kofod-Olsen, Christian K. Holm, Jesper Melchjorsen, David G. Jensen, Anne Louise Hansen, Louise B. Jørgensen, Lars Ostergaard, Martin Tolstrup, Carsten S. Larsen, Søren R. Paludan, Martin R. Jakobsen, Trine H. Mogensen

**Affiliations:** 1 Department of Infectious Diseases, Aarhus University Hospital Skejby, Aarhus, Denmark; 2 Department of Biomedicine, Aarhus University, Aarhus, Denmark; 3 Aarhus Research Center for Innate Immunology, Aarhus, Denmark; 4 Department of Virology, The State Serum Institute, Copenhagen, Denmark; George Mason University, United States of America

## Abstract

HIV infects key cell types of the immune system, most notably macrophages and CD4+ T cells. Whereas macrophages represent an important viral reservoir, activated CD4+ T cells are the most permissive cell types supporting high levels of viral replication. In recent years, it has been appreciated that the innate immune system plays an important role in controlling HIV replication, e.g. via interferon (IFN)-inducible restriction factors. Moreover, innate immune responses are involved in driving chronic immune activation and the pathogenesis of progressive immunodeficiency. Several pattern recognition receptors detecting HIV have been reported, including Toll-like receptor 7 and Retinoic-inducible gene-I, which detects viral RNA. Here we report that human primary T cells fail to induce strong IFN responses, despite the fact that this cell type does express key molecules involved in DNA signaling pathways. We demonstrate that the DNA sensor IFI16 migrates to sites of foreign DNA localization in the cytoplasm and recruits the signaling molecules stimulator of IFN genes and Tank-binding kinase, but this does not result in expression of IFN and IFN-stimulated genes. Importantly, we show that cytosolic DNA fails to affect HIV replication. However, exogenous treatment of activated T cells with type I IFN has the capacity to induce expression of IFN-stimulated genes and suppress HIV replication. Our data suggest the existence of an impaired DNA signaling machinery in T cells, which may prevent this cell type from activating cell-autonomous anti-HIV responses. This phenomenon could contribute to the high permissiveness of CD4+ T cells for HIV-1.

## Introduction

Human immunodeficiency virus (HIV) is a human retrovirus and the cause of acquired immune-deficiency syndrome (AIDS) with an estimated 34 million people infected worldwide and responsible for 1.8 million deaths annually [1], [2]. The natural history of HIV infection if left untreated is the development of progressive immunodeficiency and susceptibility to a wide range of opportunistic infections. Chronic immune activation plays a pivotal role in driving the immunopathology, and several mechanisms have been proposed to contribute to this process, including bacterial translocation from damaged gut intestinal mucosa, activation-induced death of infected and un-infected T cells, and innate recognition and pro-inflammatory responses evoked by sensing of viral replication intermediates or opportunistic pathogens [3]–[10]. Whereas much is known about the role of T cells and adaptive immunity during HIV infection, interactions between HIV and the innate immune system has only more recently been recognized to play a central part in the pathogenesis of HIV infection [6], [7]. Early innate sensing of HIV infection and activation of antiviral responses, most notably type I interferon (IFN) production, may serve a protective role for the host by restricting viral replication and spread [11]. On the other hand, it is well established that innate immune responses with secretion of cytokines and IFN contribute to chronic immune activation, the development of immunodeficiency, and progression to AIDS [6], [7].

HIV infects CD4+ cells expressing at least one of the chemokine receptors CCR5 or CXCR4, i.e. CD4+ T cells, macrophages, and dendritic cells (DC)s [12]. However, the characteristics of HIV-1 infection in different cell types are distinct in terms of permissiveness, replication rate, and cytopathic effects. In macrophages, CCR5 is the primary co-receptor and only viral strains with high affinity to CCR5 infect macrophages [13]. Furthermore HIV-1 replicates at low levels with relatively slow kinetics and induces only limited or moderate cytopathic effects [14], [15]. In contrast, HIV-1 infection in activated T cells is generally productive and cytopathic, whereas resting T cells harbor the virus in an inactive stage, although with the potential to activate viral replication upon T cell stimulation [14]–[18]. Much focus has been on the role of cellular restriction factors, most of which are IFN-stimulated genes controlling HIV replication at different stages of the replication cycle [19]–[23]. APOBEC proteins are cytidine deaminases that inhibit HIV replication in non-permissive cells by inducing cytidine to uridine editing ultimately resulting in hypermutation as well as replication defects and diminished reverse transcription [24], [25]. In contrast, tetherin works by preventing release of nascent viral particles from the cellular membrane [26]. More recently, a mechanism has been described, whereby SAMHD1 regulates cellular levels of dNTPs available, thereby restricting the replication of HIV in certain cell types [22], [27]. Finally, the Schlafen protein SLFN11 was been attributed a role as restriction factor mediated by inhibition of HIV protein synthesis [23]. Taken together, cell type differences in supporting HIV infection may be explained by variations in expression levels of restriction factors as well as by differences in innate cell-autonomous anti-HIV responses.

A prerequisite for induction of antiviral responses is the sensing of invading pathogens. Pattern recognition receptors (PRRs) are sensors of the innate immune system recognizing evolutionary conserved structures on pathogens termed pathogen associated molecular patterns (PAMP)s [28]. Activation of PRRs stimulates transcriptions factors resulting in the production of pro-inflammatory cytokines and type I IFN responses [3], [29]. The families of PRRs include the membrane-associated Toll-like receptors (TLR)s and C-type lectin receptors, as well as the cytosolic RIG-like receptor (RLR)s and Nod-like receptors [29]. For instance TLR9 detects foreign and endogenous DNA in endosomal compartments [30]. In addition, cytosolic DNA is now also recognized to be a potent stimulator of innate immunity [31], and several DNA sensors have been identified and demonstrated to be expressed in many cell types. These include DNA-dependent activator of IFN-regulatory factors (DAI), IFN inducible protein 16 (IFI16), DEAD-box polypeptide 41 (DDX41), absent in melanoma 2 (AIM2), DNA-dependent protein kinase, and most recently, cyclic guanosine monophosphate-adenosine monophosphate synthase (cGAS) [32]–[37]. DNA-induced IFN responses have been reported to proceed through a pathway involving stimulator of IFN genes (STING), TANK-binding kinase (TBK1), and IFN regulatory factor (IRF) 3 and 7 [33], [34], [38]. The expression of PRRs, including DNA sensors, is cell type specific and may also be modulated during infection, thus contributing to the specificity and timing of innate immune responses in different tissues and phases of infection [39].

During HIV infection, several nucleic acid structures are present in the cell and potentially available for recognition by cellular PRRs. This includes genomic positive strand ssRNA, RNA-DNA hybrids, ssDNA, and dsDNA [40]. PRRs are expressed in many cell types of relevance for HIV infection, and to date, several different classes of PPRs have been demonstrated to recognize HIV-derived PAMPs in vitro. First, guanine-uridine-rich ssRNA derived from HIV is recognized by TLR7/8 and stimulates plasmacytoid DCs (pDC)s and macrophages to secrete IFNα and pro-inflammatory cytokines [41], [42], and also increases cytotoxicity and IFNγ production from natural killer cells [43]. Moreover, interaction between HIV gp120 and DC-SIGN induces phosphorylation of NF-κB, and this signal from DC-SIGN appears to be required for elongation of viral transcripts and hence for synthesis of complete transcripts and productive infection [44]. Within the cytoplasm, RIG-I has been proposed to recognize HIV genomic ssRNA [45], [46]. In addition, the viral capsid constitutes a PAMP and is recognized by several cytosolic sensors, including Cyclophilin A and TRIM5α [47], [48]. Finally, a role for DNA sensors in HIV recognition has been suggested by a number of studies as described below [49], [50].

A seminal study by Liebermann and associates demonstrated that HIV-1 escapes innate recognition and IFN production in a manner dependent on the cytosolic exonuclease Trex1, which degrades cytosolic HIV DNA, and thereby prevents recognition of HIV DNA [49]. This is supported by a study by Greene and associates, in which abortive HIV infection and accumulating replication intermediates, such as HIV DNA, was demonstrated to activate a host defense program involving pro-apoptotic and pro-inflammatory responses [51]. Indirect suggestions of the existence of DNA sensing during HIV infection has been provided by Lepelley et al. who demonstrated enhanced sensing of HIV-infected cells as compared to cell-free HIV virions in pDCs by a TLR7-dependent mechanism [50]. Importantly, this study also clearly indicated a role for TLR7-independent cytosolic HIV sensing, with DNA sensors representing potential candidates. Most recently, studies by us and others have demonstrated a role for IFI16 and cGAS in recognition of HIV DNA and induction of IFN responses in macrophages [52], [53]. However, the role and function of DNA sensors in T cells remains largely unknown. Although generally regarded as cells of the adaptive immune system, T cells do express a wide range of PRRs, including intracellular nucleic acid sensors, such as IFI16 [54], [55].

HIV-derived molecules are not the only source of PAMPs that may influence HIV replication during infection. Several studies have shown that opportunistic pathogens can also modulate the replication of HIV [6]–[10]. Mechanistically, pathogens stimulate viral replication by activating the cellular transcriptions factor NF-κB, which has the ability to bind to the HIV long terminal repeat promoter region, thereby inducing viral replication [56], [57]. In contrast, other pathogens may negatively regulate HIV replication by activating a strong type I IFN response with up-regulation of cellular restriction factors and other IFN-stimulated genes (ISG)s [57]–[59]. However, the possible effects triggered by sensing of DNA derived from HIV replication, from bacterial translocation or from opportunistic pathogens has received only little attention.

The aim of this study was to evaluate the ability of T cells to sense DNA produced during the HIV-1 replication cycle or DNA derived from other pathogens, to characterize the down-stream response, and to describe the possible effects on HIV replication. Here we demonstrate that introduction of synthetic DNA into the cytoplasm of activated T cells does not affect the replication of HIV and does not lead to significant induction of IFN responses. This is in contrast to the situation in primary macrophages and THP-1 cells, in which DNA sensing induces ISGs and inhibits HIV replication. We show that activated T cells detect intracellular DNA through IFI16 and recruits STING and TBK1. However, the detection does not impact the level of expression of IFN and ISGs. By contrast, we show that the T cells do have the capacity to evoke IFN responses through the RIG-I pathway. These findings lead us to suggest that the DNA-activated signaling pathway inducing IFN responses is non-functioning in activated T cells, and we propose that this feature may be a contributing factor to the permissiveness of T cells to HIV replication.

## Results

### Cytosolic DNA fails to induce antiretroviral effects in IL2/PHA-stimulated PBMCs

**Figure 1:**
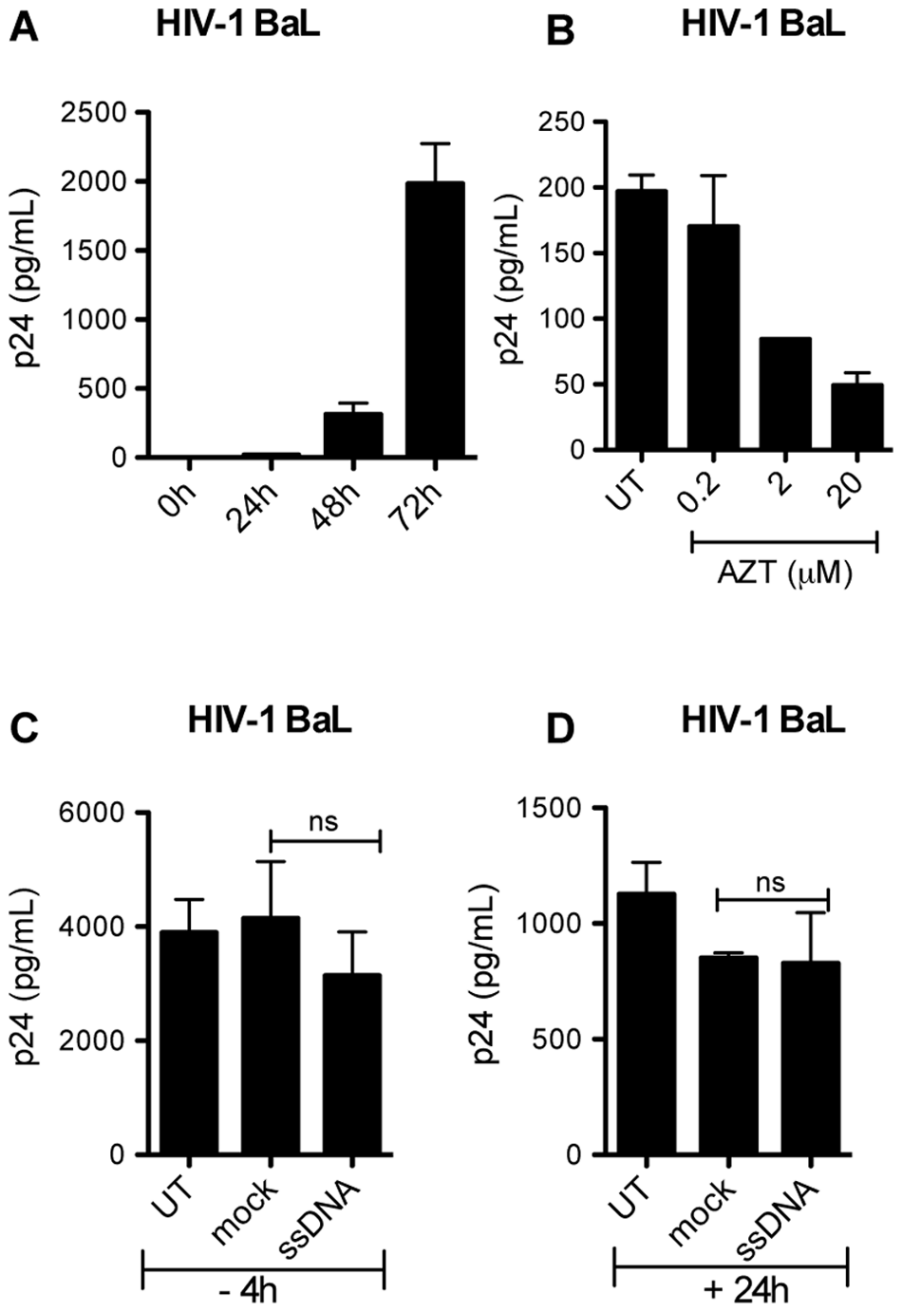
Cytosolic DNA does not affect HIV-1 BaL replication in IL2/PHA PBMCs. (**A**) IL2/PHA PBMCs were infected with HIV-1 BaL at an MOI of 0.002, and p24 levels were measured in the supernatants after 24, 48, and 72 hours of infection. (**B**) IL2/PHA PBMCs were pretreated with AZT at increasing doses 30 min before infection with HIV-1 BaL at an MOI of 0.002. Levels of p24 were measured in the supernatants 72 hours post infection by ELISA. (**C, D**) IL2/PHA PBMCs were transfected with ssDNA (2 µg/mL) (**C**) 4 hours before or (**D**) 24 hours after infection with HIV-1. Supernatants were harvested 72 hours post infection and p24 levels measured by ELISA. Data are shown as mean of triplicates +/− SD. Similar results were obtained in three or more independent experiments. Mock, Lipofectamine.

Previous studies have shown that immune activation triggered by co-infecting pathogens can affect the replication of HIV [56], [57]. Several reports suggest that HIV-derived DNA generated during the viral replication cycle may be recognized by PRRs in the cytosol, at least in the absence of the DNA exonuclease TREX1 [52], [53], [60], [61]. In the present study, we wanted to evaluate the effects of DNA recognition on HIV replication in activated T cells. For this purpose, PBMCs were harvested from healthy donors and the non-adherent subpopulation was activated with IL2 and PHA for 48 hours and then IL2 alone for 24 hours before infection with HIV-1 BaL. Following IL2/PHA stimulation, the PBMCs consisted mainly of activated T cells expressing high levels of HLA-DR, CD38, CD25 and CD69 (Figure S1). The IL2/PHA-stimulated PBMCs were infected with HIV-1 BaL at an MOI of 0.002 in the presence of IL2. HIV p24 was detectable in the supernatants 48 hours after infection, and levels were further increased at 72 hours (Figure 1A). Moreover, viral replication could be inhibited in a dose dependent manner by using the antiretroviral compound AZT (Figure 1B).

For DNA transfections, we used a ssDNA sequence derived from the 5′ untranslated region (5′UTR) covering the TAR-loop of the HIV genome [53]. When introducing ssDNA into the cytoplasm by transfection either 4 hours before, or 24 hours after infection with HIV-1 BaL, we observed none or only very modest effects on the replication of HIV, as measured by the levels of p24 in the supernatants by ELISA 72 hours post infection (Figure 1C–D). Based on these data we conclude that cytosolic DNA fails to stimulate antiretroviral activity to a measurable extent in activated T cells.

### Cytosolic DNA does not induce type I IFN and pro-inflammatory cytokines in IL2/PHA PBMCs

Next, we investigated the mechanism accounting for the inability of DNA transfection to affect HIV replication. In addition to ssDNA we also included a 60-mer dsDNA, which has previously been demonstrated to be recognized by the DNA sensor IFI16 and to activate IFN and ISGs [33]. In order to characterize the response to cytosolic DNA, we transfected IL2/PHA PBMCs with either ssDNA or dsDNA. By using FITC-labeled ssDNA, the transfection efficiency was estimated to be around 30 % depending on the donor and the individual experiment as measured by both flow cytometry and confocal microscopy (Figure S2 and data not shown). Moreover, ssDNA and dsDNA were transfected with comparable efficiency (data not shown). These data demonstrate that although T cells are known to be difficult to transfect and to achieve protein expression and siRNA-mediated knock-down of gene expression, DNA can be delivered into these cells, hence allowing assessment of immune activation by this PAMP.

**Figure 2:**
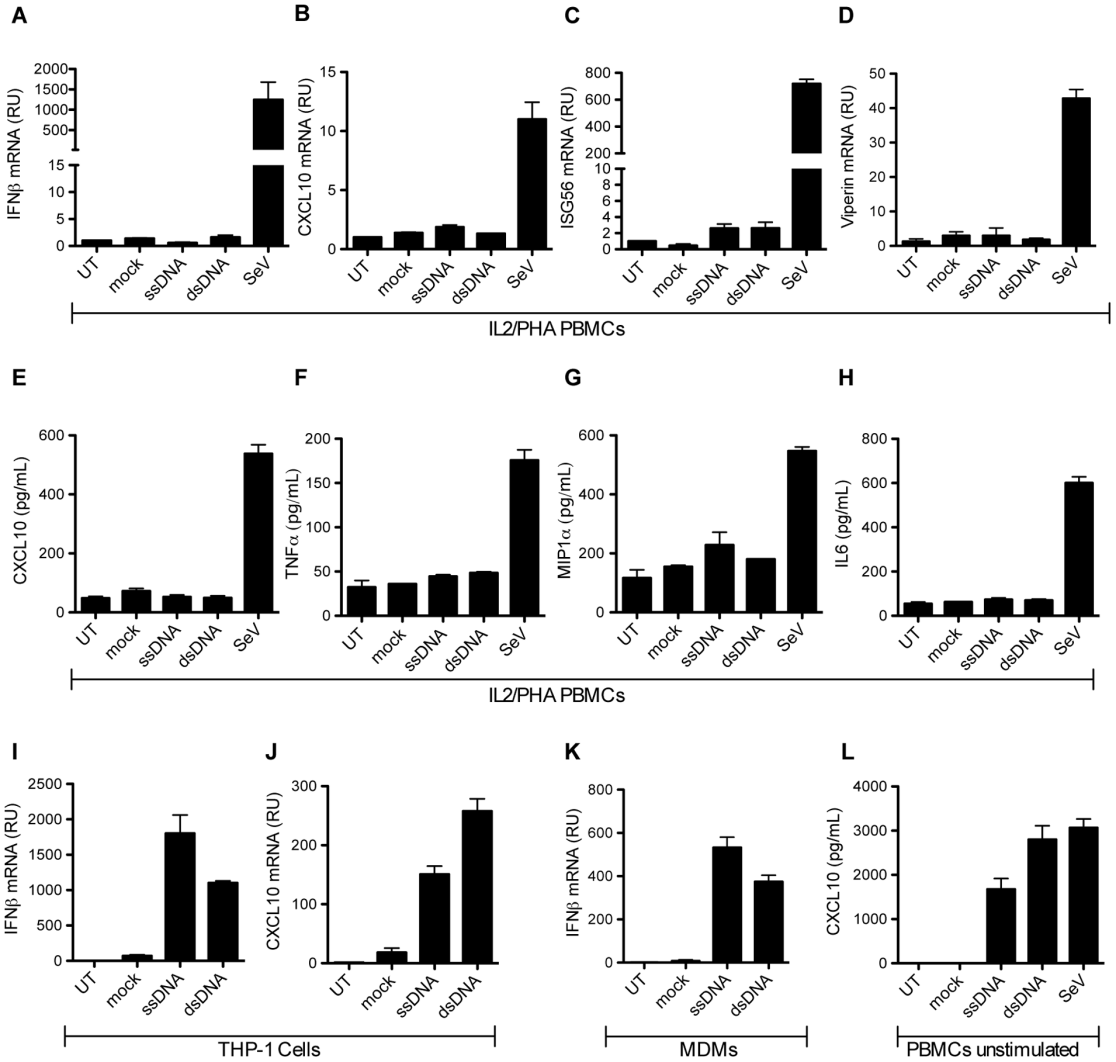
IL2/PHA PBMCs fail to induce type I IFN responses and pro-inflammatory cytokines upon DNA transfection. (**A–D**) IL2/PHA PBMCs were transfected with HIV-derived ssDNA and dsDNA (2 µg/mL), or infected with SeV (MOI 0.5). Total RNA was isolated after 6 hours for RT-qPCR measurements of (**A**) IFNβ, (**B**) CXCL10, (**C**) ISG56, and (**D**) Viperin. (**E–H**) IL2/PHA PBMCs were transfected with ssDNA and dsDNA (2 µg/mL), or infected with SeV (MOI 0.5). Supernatants were harvested after 24 hours and analyzed for CXCL10, TNFα, MIP-1α, and IL6 protein levels. (**I–J**) PMA (100 nM) treated THP-1 cells were transfected with ssDNA and dsDNA (2 µg/mL). Total RNA was harvested after 6 hours for RT-qPCR measurements of IFNβ and CXCL10 mRNA. (**K**) Primary human monocyte-derived macrophages (MDM)s were transfected with ssDNA and dsDNA (2 µg/mL). Total RNA was harvested after 6 hours for RT-qPCR measurements of IFNβ. (**L**) Un-stimulated PBMCs were isolated and immediately transfected with ssDNA and dsDNA (2 µg/mL), or infected with SeV (MOI 0.5). Supernatants were harvested after 24 hours and analyzed for CXCL10 by ELISA. Both PCR and ELISA data are shown as means of triplicates +/− SD. Similar results were obtained in three independent experiments. Mock, Lipofectamine.

In order to measure induction of IFNs, ISGs, and pro-inflammatory cytokines were evaluated on RNA isolated 6 hours post transfection by RT-qPCR and by ELISA on culture supernatants harvested 24 hours post treatment (Figure 2). Surprisingly, neither of the DNA oligos induced significant amounts of IFNs, ISGs, nor pro-inflammatory cytokines in IL2/PHA PBMCs (Figure 2A–H). Only minor induction of ISG56 and MIP1α was observed (Figure 2C and 2G) after transfection with ssDNA, and these were not statistically significant. A more detailed analysis of the kinetics of gene expression revealed that no or only very limited IFNβ and CXCL10 mRNA was induced between 4 and 24 h post stimulation (data not shown). In contrast, SeV, which induces innate immune responses via the RIG-I pathway [62], induced high levels of IFNs, ISGs, and pro-inflammatory cytokines in IL2/PHA PBMCs (Figure 2A–H). In addition, the TLR9 agonist ODN2216, also evoked expression of CXCL10 in the activated T cells (Figure S3). These data suggest that the IL2/PHA PBMCs selectively are unresponsive to cytosolic DNA rather than having a general defect in innate antiviral pathways.

In THP-1 cells differentiated into a macrophage-like phenotype in the presence of PMA, both ssDNA and dsDNA induced high levels of the ISG CXCL10 (Figure 2I–J). Similar inductions were observed for ISG56 in THP-1 cells (data not shown). Importantly, primary human MDMs did respond to cytosolic ssDNA or dsDNA by mounting a vigorous IFN response (Figure 2K). Finally, we used un-stimulated PBMCs, which were also found to induce high levels of CXCL10 in response to DNA transfection (Figure 2J). Thus, IL2/PHA-activated PBMCs, mainly consisting of activated T cells, fail to induce strong innate immune responses after stimulation with intracellular DNA. This is in striking contrast of other cells of the myeloid cell lineage, most notably MDMs.

### Neither un-stimulated nor IL2/PHA stimulated CD4+ T cells respond to DNA transfection

**Figure 3:**
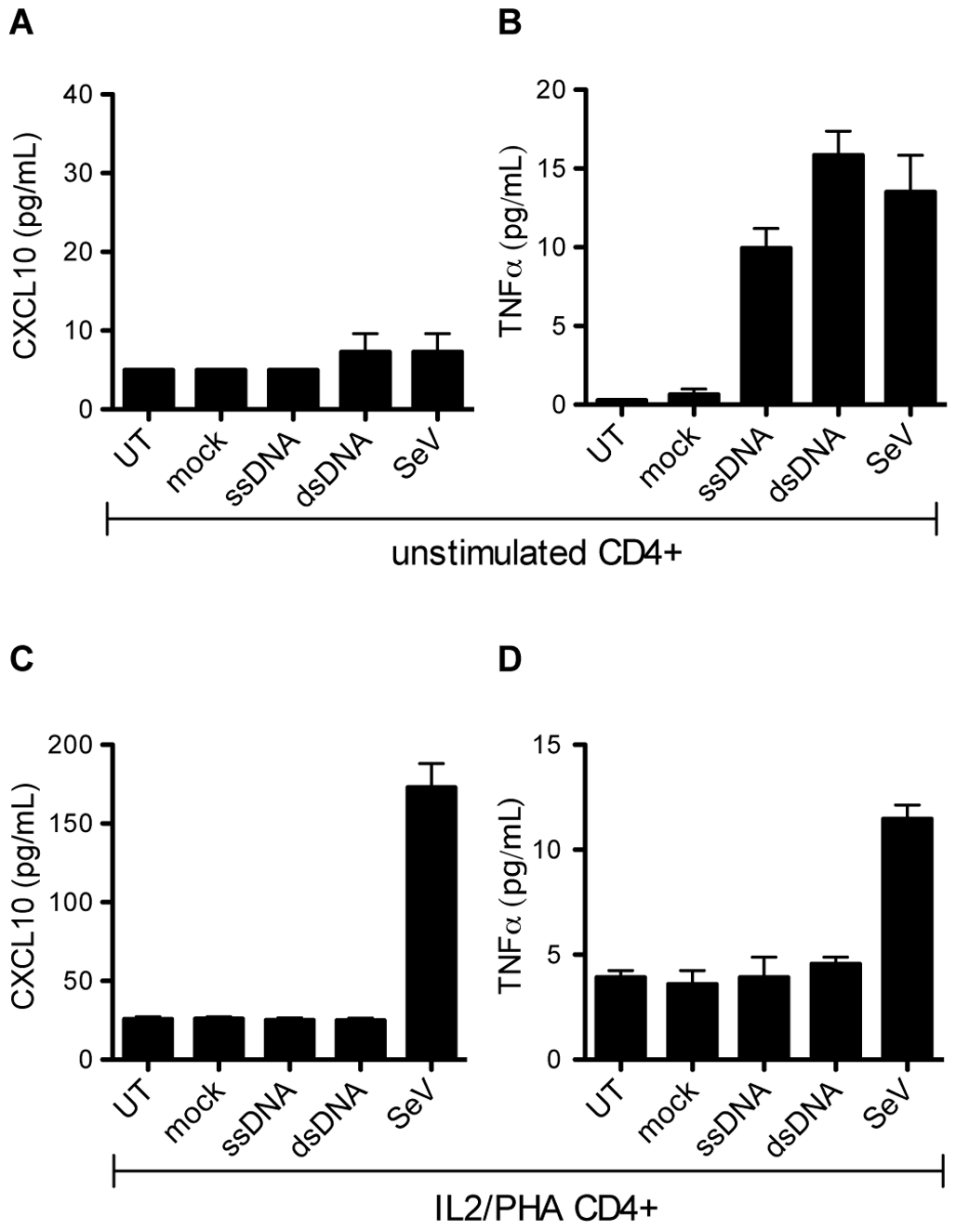
Comparison of un-stimulated and IL2/PHA stimulated CD4+ cells in response to DNA transfection. CD4+ T cells were isolated and either left untreated or activated with IL2/PHA prior to further treatment. The cells were transfected with ssDNA and dsDNA (2 µg/mL), or infected with SeV (MOI 0.5). Supernatants were harvested 24 hours later and analyzed for levels of (**A, C**) CXCL10 and (**B, D**) TNFα. Data are shown as means of triplicates +/− SD. Similar results were obtained in two independent experiments. Mock, Lipofectamine.

After documenting that IL2/PHA PBMCs are unresponsive to DNA, we aimed to examine if this also applies to purified T cells. To address this issue, we selected CD4+ cells from freshly isolated PBMCs. Purity of CD4 selection was verified by flow cytometry, where 98% of the cells were found to be CD4+ (Figure S4). The CD4+ cells were either stimulated with IL2/PHA or left un-stimulated prior to transfection. Activation markers on these isolated CD4+ IL2/PHA stimulated cells were evaluated by flow cytometry staining for CD25 and CD69, confirming that the cells were highly activated (Figure S4). Upon DNA transfection, we observed that neither un-stimulated nor IL2/PHA stimulated CD4+ cells induced significant levels of CXCL10 (Figure 3A, C). Furthermore, resting CD4+ T cells did not produce CXCL10 in response to Sendai virus infection, whereas the IL2/PHA-activated CD4+ T cells responded to this infection in a manner similar to the IL2/PHA-activated PBMCs. Regarding TNFα, we did observe minor induction after transfection with ssDNA and dsDNA in un-stimulated CD4+ cells, but not in IL2/PHA treated cells (Figure 3B, D).

In summary, these results demonstrate that neither IL2/PHA-activated PBMCs nor pure CD4+ T cells (activated or unactivated) stimulated the IFN/ISG pathway in response to intracellular DNA, although low levels of TNFα expression were induced in un-stimulated CD4+ cells. This suggests that the lack of innate immune response observed in IL2/PHA PBMCs does not represent an artifact due to the IL2/PHA stimulation conditions, but rather reflects a weak response to DNA in T cells in general.

### Presence of cytoplasmic DNA does not induce pro-apoptotic pathways in IL2/PHA PBMCs

It has been demonstrated that accumulation of incomplete HIV-1 reverse transcripts, including DNA intermediates, induce a caspase-dependent pro-apoptotic response in abortively infected CD4+ T cells from human lymphoid tissue [51]. It was proposed that this mechanism may be a leading course of the massive loss of CD4 cells during HIV infection [51]. We therefore wanted to examine if transfection of HIV DNA into IL2/PHA PBMCs induced pro-apoptotic pathways, hence potentially explaining the lack of IFNs and pro-inflammatory cytokines observed in the present study. For this purpose, we used a flow cytometry-based assay to measure activation of the pro-apoptotic caspases 3, 7, 8, and 9, as well as dead cells. We transfected IL2/PHA activated PBMCs with ssDNA and evaluated caspase activity and cell death after 4 and 24 hours. However, we observed no activation of caspases and also no stimulation of cell death by the DNAs 24 h post transfection as compared to mock-transfected cells (Figure S5). By contrast, Etoposide, which is an antitumor drug causing DNA damage, induced high level of caspase activity and cell death as expected. Similar results were obtained when examining the cells 4 hours post transfection (data not shown). Hence, we conclude that in our experimental set-up, the presence of cytoplasmic HIV DNA does not induce apoptosis nor activation of caspase 3, 7, 8 or 9.

### The DNA sensor IFI16 as well as the downstream signaling molecules STING, TBK1, and IRF3, are expressed in IL2/PHA-stimulated PBMCs

**Figure 4:**
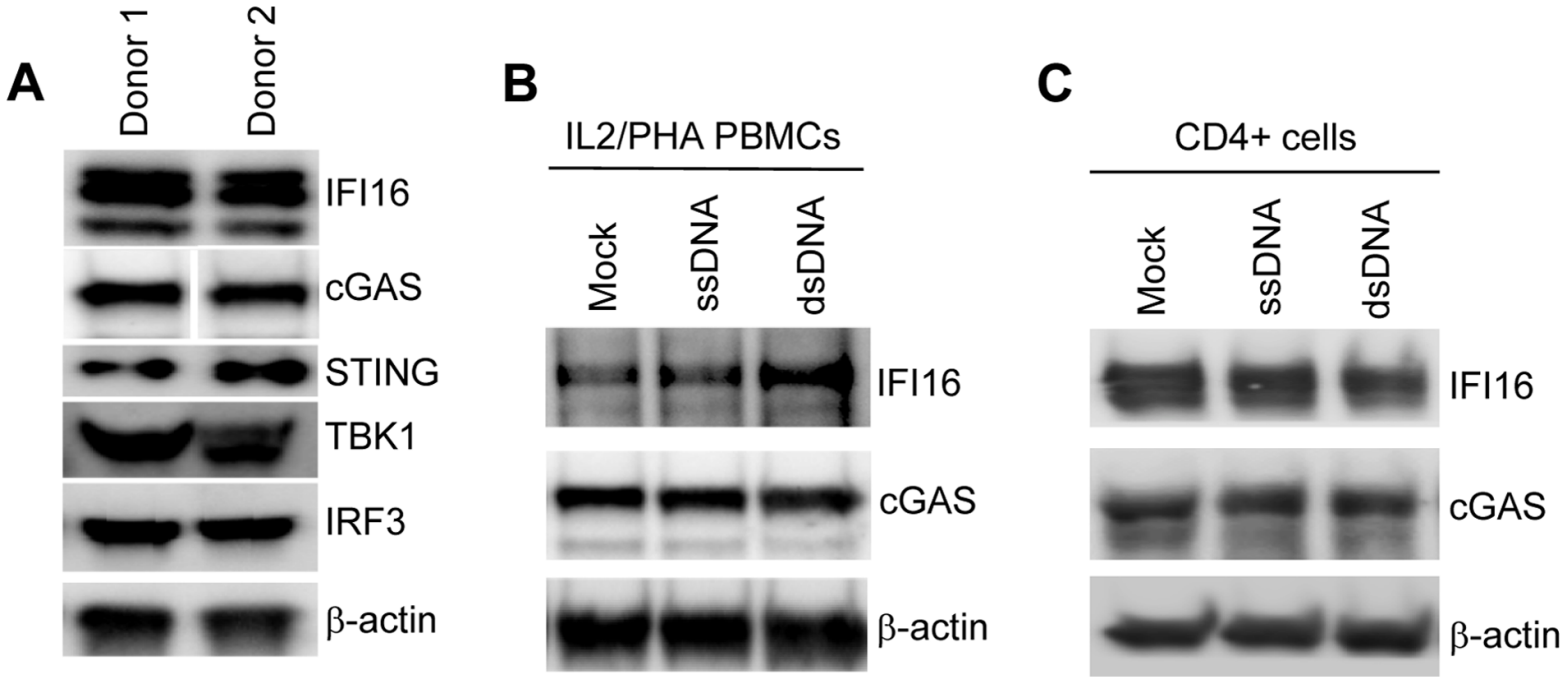
Expression of DNA signaling pathway molecules in IL2/PHA PBMCs and CD4+ T cells. (**A**) Whole cell lysates of IL2/PHA PBMCs from 2 donors were analyzed for expression of IFI16, STING, TBK1, IRF3 and β-actin by Western Blotting. (**B, C**) Whole cell lysates from IL2/PHA-treated PBMCs and CD4+ T cells were stimulated with ssDNA, dsDNA (both 2 µg/mL) or lipofactamine for 2 h of IL2/PHA PBMCs were analyzed for levels of IFI16 and cGAS by Western Blotting. Similar results were obtained with two independent donors.

Based on our observation of only very modest inductions of type I IFNs, ISGs and cytokines in activated PBMCs in response to DNA transfection, we were interested in examining whether the proteins in the IFN/ISG-inducing pathway were expressed in these cells. To address this issue we harvested cell lysates and analyzed for the presence of IFI16, cGAS, STING, TBK1, and IRF3 by Western blotting. As shown in Figure 4A, the main components of the cellular DNA sensing and signaling pathway were expressed in IL2/PHA PBMCs, despite the absence of DNA-inducible IFN/ISG responses. The levels of expression of IFI16 and cGAS in the IL2/PHA PBMCs were not affected by the DNA stimulation (Figure 4B). In addition, we found that a similar pattern of expression of DNA sensors was found in purified CD4+ T cells (Figure 4C), hence eliminating the possibility that the observed expression of IFI16 and cGAS in the IL2/PHA PBMCs was due to contaminating myeloid cells.

### Transfected DNA co-localizes with IFI16 in T cells and recruits TBK1 to STING foci

**Figure 5:**
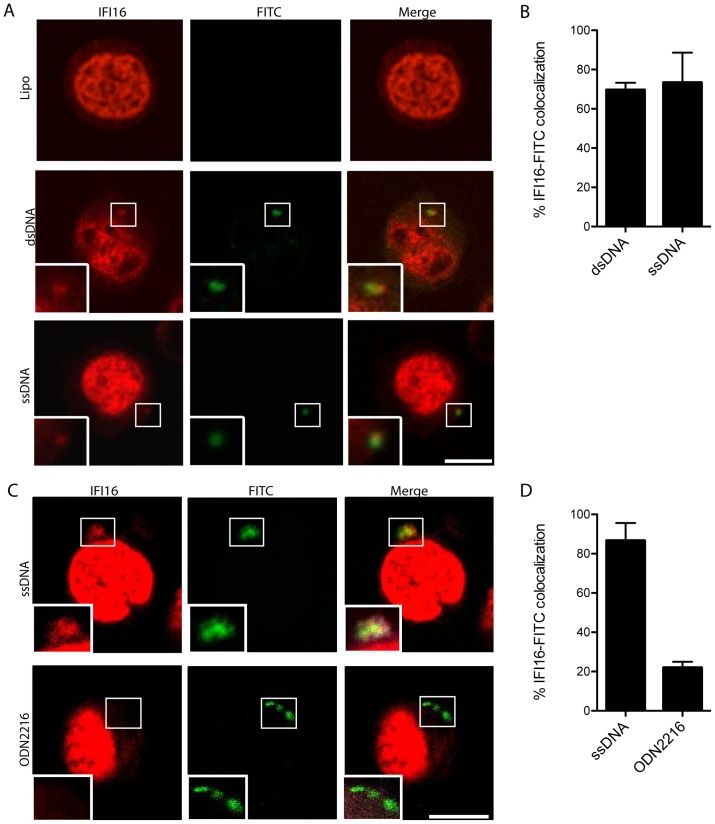
Transfected DNA co-localizes with the DNA sensor IFI16 in activated T cells. (**A, C**) IL2/PHA PBMCs transfected with 2 µg/mL of FITC-labeled DNA or 0.5 µM ODN2216 as indicated for 2 hours were fixed and stained with anti-IFI16 antibody and visualized by confocal microscopy. Presented cells stained positive for CD3. IFI16 is shown in red and DNA in green. (**B, D**) Percentage colocalization of cytoplasmic spots positive for IFI16 and DNA. Data is based on quantification of IFI16/DNA spots in more than 100 cells per donor in 3 different donors. Data is shown as means +/− SD. Scale bar, 5 µm.

For further investigation of DNA recognition in T cells, we used ssDNA and dsDNA both labeled with FITC, and transfected these DNA oligos into IL2/PHA PBMCs. The cells were stained with antibodies for CD3 (Figure S6) and IFI16, and analyzed by confocal microscopy. As illustrated in Figure 5A, in CD3+ cells, ssDNA and dsDNA co-localized with IFI16 in the cytosol. For quantification of the co-localization, more than 100 FITC-positive spots were counted for each DNA species and percentage of IFI16-FITC co-localization was evaluated. We found that about 75 % of the DNA spots containing either ssDNA or dsDNA also stained positive for IFI16 (Figure 5B). Importantly, the strong association between DNA and IFI16 was not observed for all types of DNA, since the TLR9 agonist ODN2216 exhibited minimal colocalization with IFI16 (Figure 5C and 5D).

**Figure 6:**
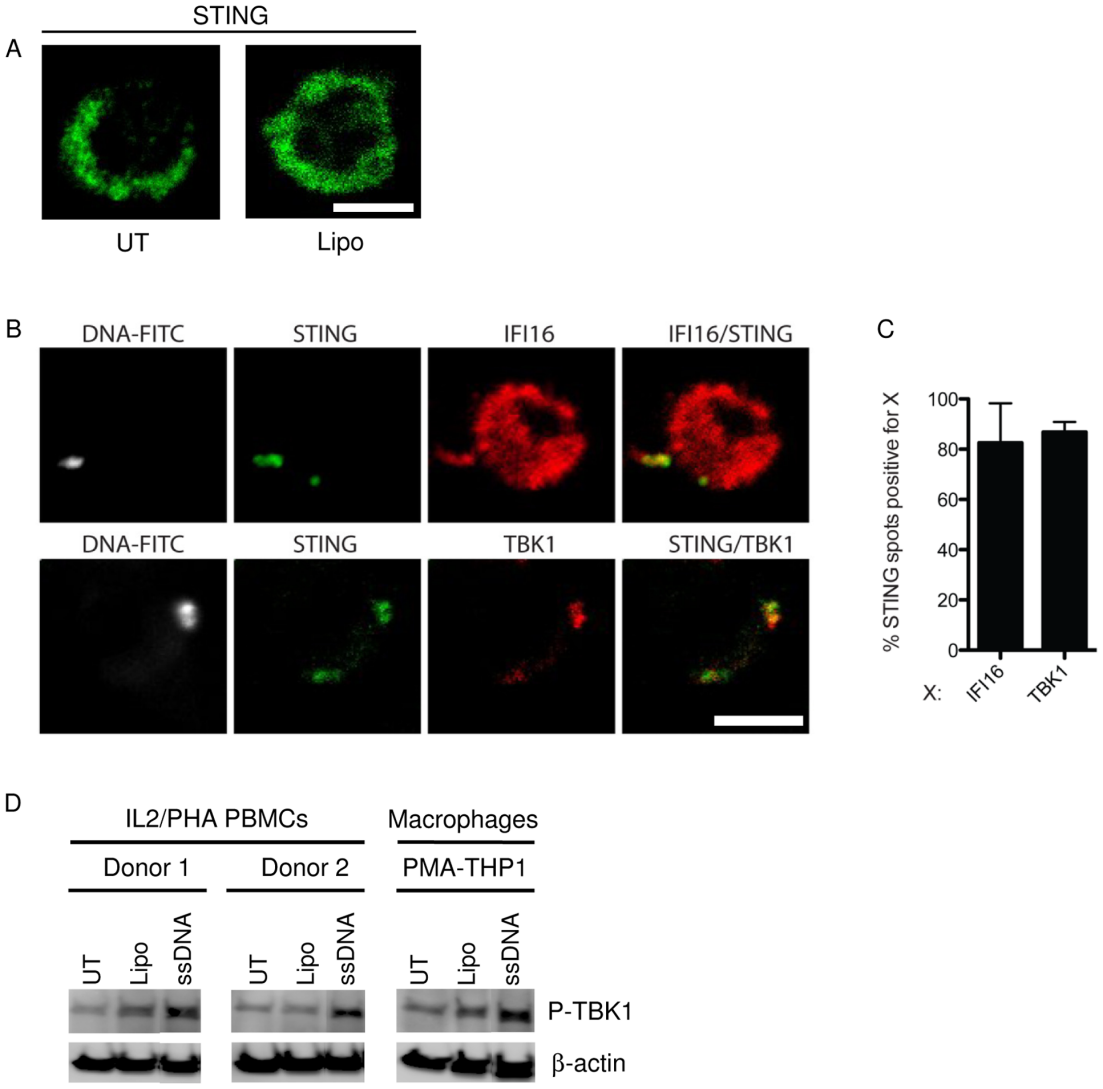
Transfected DNA co-localizes with STING and TBK1. (**A**) IL2/PHA PBMCs were left untreated or treated with Lipofectamine2000 for 2 hours prior to fixation and staining with anti-STING antibody (Green). (**B**) IL2/PHA PBMCs transfected with 2 µg/mL of FITC-labeled DNA as indicated for 2 hours were fixed and stained with anti-IFI16 and anti-STING antibodies (upper panel) or anti-STING and anti-TBK1 antibodies (lower panel) and visualized by confocal microscopy. STING is shown in green, IFI16 and TBK1 in red, and DNA in white. (**C**) Percentage co-localization of cytoplasmic spots positive for the respective staining was quantified by counting more than 100 cells per donor in 3 different donors. Data is shown as means +/− SD Scale bar, 5 µm. (**D**) Whole cell lysates of IL2/PHA PBMCs from 2 donors and PMA-differentiated THP1 cells treated with ssDNA (2 µg/mL) for 2 h were analyzed for levels of phosphorylated TBK1 by Western Blotting.

For examination of downstream signaling molecules, we also wanted to explore whether DNA sensing in T cells resulted in mobilization of the cytosolic adaptor STING, which is essential for signal transduction downstream of most DNA sensors [33], [34], [38]. In CD3+ cells not transfected with DNA, we observed that STING was distributed widely throughout the cytosol (Figure 6A), and like IFI16 did not assemble into discrete foci (Figure 5A). By contrast, DNA transfection of the T cells stimulated formation of STING foci, most of which also stained positive for IFI16 and ssDNA (Figure 6B–C). STING also co-localized with the IRF3 kinase TBK1 after DNA transfection indicating the assembly of signaling complexes (Figure 6B–C). In support of this observation, activated T cells transfected with ssDNA displayed a capacity to mediate TBK1 phosphorylation comparable to what was observed in PMA-differentiated THP1 cells receiving the same treatment (Figure 6D). Activation of the NF-κB pathway was examined by measurement of phospho-IκBα in samples stimulated with ssDNA for, 1, 2, or 4 hours. This analysis revealed that the NF-κB pathway was not activated by DNA in activated T cells (data not shown).

Taken together, these data demonstrate that activated T cells react to intracellular DNA by mobilization of key recognition and signaling molecules into specific subcellular areas, hence indicating assembly of a signaling complex. Despite this, DNA does not induce significant IFN responses in activated T cells in contrast to the situation in MDMs.

### IL2/PHA PBMCs are responsive to type I IFN, which inhibits HIV replication

**Figure 7:**
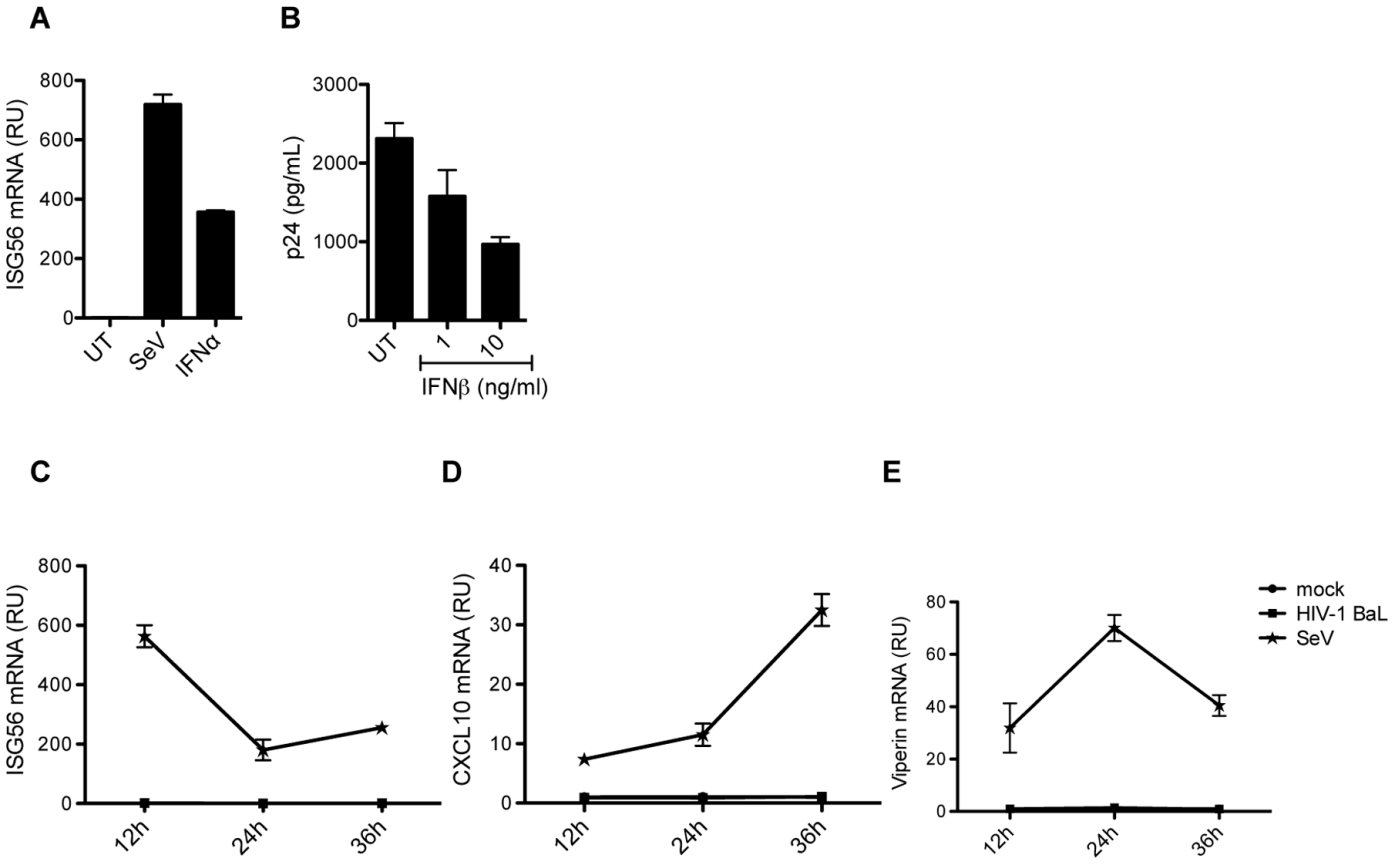
Type I IFN induces ISGs and inhibits HIV-1 BaL replication in IL2/PHA PBMCs. (**A**) IL2/PHA PBMCs were infected with SeV (MOI 0.5) or treated with IFNα (20 ng/mL). Total RNA was harvested after 6 hours for RT-qPCR measurements of ISG56 mRNA. (**B**) IL2/PHA PBMCs were pre-treated with IFNβ in increasing doses 16 hours prior to infection with HIV-1 Bal (MOI 0.002). Levels of p24 were measured in the supernatants 72 hours post infection. (**C–E**) IL2/PHA PBMCs were infected with HIV-1 BaL (MOI 0.002) or SeV (MOI 0.5) or mock infected. Total RNA was harvested after 12, 24, and 36 hours for RT-qPCR measurements of (**C**) ISG56, (**D**) CXCL10, and (**E**) viperin mRNAs.

Finally, we were interested in examining whether exogenously added type I IFN was able to restrict HIV-1 replication in activated T cells in our experimental set-up. Stimulating the IL2/PHA PBMCs with type I IFN did induce strong ISG expression, hence demonstrating the IFN responsiveness of these cells (Figure 7A). We next wanted to examine whether IFN treatment could affect the replication of HIV in IL2/PHA PBMCs. As demonstrated in Figure 7B, type I IFN treatment did indeed inhibit viral replication in a dose dependent manner, although HIV replication could not be totally abolished (Figure 7B). To test whether replication competent HIV-1 induces ISG responses response in IL2/PHA PBMCs, we infected the cells with HIV-1 BaL, and harvested RNA at 12, 24, and 36 hours post infection. As for ssDNA, HIV-1 BaL did not induce expression of the two ISGs ISG56 and CXCL10 in IL2/PHA PBMCs (Figure 7C, D). Collectively, these data demonstrate that activated T cells do respond to type I IFNs, which have anti-retroviral activity in these cells. However, activated T cells fail to induce IFN/ISG responses through the DNA sensing pathway or following HIV infection, hence preventing this cell type from activating a potential cell-autonomous anti-HIV pathway.

## Discussion

Innate sensing of invading pathogens plays a major role in early restriction and containment of microbial infections and is also a prerequisite for the activation and maturation of adaptive immune responses and the generation of immunity [39]. During recent years it has been appreciated that the innate immune system contributes to early antiviral defense against HIV as well as to the pathogenesis of both acute and chronic HIV infection [63]. Several cellular PRRs sensing HIV PAMPs have been identified [64]. Most recently, HIV DNA has been demonstrated to be able to stimulate antiviral IFN responses [52], [53], [60], [61]. Although HIV can infect macrophages, CD4+ T cells are the main target cell supporting HIV replication and allowing the establishment of productive infection to drive the pathogenesis of the disease. Despite this, knowledge on PRRs recognizing HIV PAMPs in T cells remains very sparse. Therefore, we were interested in characterizing the DNA sensing machinery in activated T cells and to describe the impact of cellular DNA stimulation on HIV replication.

Here, we demonstrate that delivery of exogenous DNA into the cytoplasm of activated T cells does not affect the replication of HIV-1 BaL, irrespective of whether DNA is introduced prior to or post infection. At the mechanistic level, we demonstrate that cytosolic HIV-derived DNA does not induce type I IFN nor ISGs in activated T cells in striking contrast to the responses observed in macrophages. We further show that activated T cells detect intracellular HIV DNA through the DNA sensor IFI16 as evaluated by co-localization of DNA and IFI16 and mobilization of the downstream signaling molecules STING and TBK1. Despite this, HIV DNA fails to activate type I IFN responses, pro-inflammatory cytokines or apoptosis. Our data suggest that the inability of activated T cells to produce IFN in response to DNA may play a role in the permissiveness of this cell type to HIV infection.

The main finding of the present study is that cytosolic HIV DNA fails to induce IFN responses in T cells despite the expression of IFI16, co-localization between IFI16 and DNA and assembly of a protein complex with features of the STING signalsome, most notably recruitment of TBK1. IFI16 is a member of the PYHIN protein family and is expressed in epithelial and haematopoetic cells, including T cells [55]. IFI16 functions as an innate PRR for intracellular DNA inducing IFNβ through STING and IRF3 and possibly NF-κB [33]. We recently reported that IFI16 acts as a sensor for HIV-1 DNA in macrophages stimulating antiviral activity [53]. In the present study, we report that similar to MDMs, T cells recognize DNA through a process involving IFI16, but this cell type does not induce significant expression of IFNs, ISGs nor pro-inflammatory cytokines. Recent studies have also demonstrated a role for cGAS in innate immune activation by HIV-1 in a monocytic cell line as well as in primary human macrophages, although only when the HIV-2 gene Vpx was coexpressed [52], [53]. In the present study we focused on IFI16 and did not address a possible role for cGAS in recognition of HIV in T cells, although we did observe that this protein was expressed in the activated T cells, suggesting that absence of cGAS in T cells does not explain the lack of IFN responses. Concerning the mode of delivery it should be noted that by transfecting ssDNA into cells, a fraction of the ssDNA may be transiently associated with endosomes and possibly be recognized by TLR9. This scenario differs from natural HIV infection, in which HIV cDNA generated by reverse transcription is delivered into the cytosol, possibly protected within the viral capsid to some extent. However, based on the colocalization of transfected ssDNA with STING and TBK1 we believe that the majority of the transfected DNA in our experimental set-up was delivered to the cytosol available for sensing by cytosolic DNA sensors.

Here we have not identified the step, at which the IFN pathway is defect in activated T cells. Given that key proteins in the DNA signaling pathway are expressed in activated T cells and STING is mobilized to recruit and phosphorylate TBK1, it may be hypothesized that the block in IFN production could be at the level of the function of the STING signalsome, or alternatively at the transcriptional or epigenetic level. However, after infection with Sendai virus, where the RNA sensor RIG-I is responsible for IFN induction [62], IFN/ISGs expression was induced, demonstrating that the RIG-I pathway and downstream transcriptional machinery is fully functional in activated T cells. Based on these findings we suggest that the DNA signaling machinery is partly defective in T cells resulting in impaired ability to respond to DNA pathogens, including HIV, and to stimulate antiviral IFN responses.

The insensitivity to cytosolic DNA was observed irrespective of the timing of DNA transfection relative to infection of cells with HIV. Thus, even if DNA was present 4 hours prior to the addition of virus, this did not elicit antiviral responses capable of preventing or limiting infection. Once established, HIV replication was also not affected by DNA transfection. For other PRRs, it has previously been reported that PAMPs can both activate and inhibit HIV replication, through NF-κB and IFNs, respectively [10], [65], [66]. In the present study, cytosolic DNA induced neither NF-κB activation nor IFN production in IL2/PHA PBMCs. Studies focusing on opportunistic pathogens and the effects of PRR activation have demonstrated diverse effects of PAMPs upon HIV replication. For example, activation of TLR signaling by *Mycobacterium tuberculosis* induces HIV replication by an NFAT dependent mechanism [6]. In addition, herpes simplex virus type 1 and cytomegalovirus infection increase HIV replication, and human herpes virus type 6 induces reactivation of latently infected HIV [7]–[9]. On the other hand, *Neisseria meningitidis* infection inhibits HIV replication by inducing IFN responses through TLR9 [10]. A single study evaluated the effects of DNA sensing on replication of pseudotyped HIV in 293T cells and reported that forced expression of the DNA sensor DAI enhanced HIV replication by a mechanism dependent of NF-κB [67]. The discrepancies between those studies and the present study appear to be primarily the cell type used, since most of the above cited studies did not use infectious replication competent virus and were not performed in activated human primary T cells.

It is well-established that activated T cells are highly permissive to HIV infection, whereas resting T cells block infection at a post-entry step of the viral lifecycle [21], [22], [51]. These differences are not fully understood although mechanisms have been proposed [21]. Most notably, Baldauf et al. recently demonstrated that SAMHD1 prevents HIV reverse transcription in resting T cells by lowering the amounts of dNTPs available for reverse transcription resulting in viral restriction [22]. Supporting this conclusion, the authors also demonstrated that a mutation in SAMHD1 in cells from a patient with Aicardi-Goutiere's syndrome renders resting CD4+ T cells permissive to HIV infection [22]. The majority of the present study was performed in IL2/PHA-activated T cells in order to allow for infection with HIV in vitro. We were interested in understanding if innate immune responses to DNA may differ in T cells depending on the state of activation and thus be involved in the restricted HIV replication in resting T cells. However, like activated CD4+ T cells, un-stimulated CD4+ T cells did not induce type I IFNs in response to DNA transfection. Although we did observe some differences in TNFα expression after transfection, these minor differences in DNA responsiveness are unlikely to account for differences in HIV permissiveness between resting and activated T cells. Finally, a study on abortive HIV infection in resting CD4+ T cells from lymphoid tissue previously demonstrated caspase 1 and −3 dependent pro-apoptotic responses induced by HIV DNA intermediates [51]. In our study, which was performed primarily on highly activated T cells/PBMCs, we did not observe any apoptotic responses supporting previous data and underscoring the differences between abortive and productive infection in activated T cells.

The present work reveals significant differences between myeloid cells and T cells in responsiveness to cytosolic DNA. Whereas the monocytic cell line THP1, MDMs, and PBMCs induced strong IFN and ISG responses, we observed almost non-existent IFN and ISG induction in activated T cells in response to DNA transfection. Thus major differences exist in how monocytes/macrophages and T cells react to incoming cytosolic DNA. One implication of the existence of DNA-inducible IFN responses in monocytes and macrophages is that M tropic HIV strains with preferential infection of macrophages need to develop more sophisticated evasion strategies to be able to survive and persist in this cell type. The ability of HIV to avoid IFN induction or even to actively suppress such responses is well established [47]. Several elegant mechanisms have been identified, by which HIV is able to interfere with triggering of the IFN system, for example by depletion of the cytosolic RNA receptor RIG-I [45], or degradation of IRF3, which plays a central role in the expression of IFNs and ISGs [68]. However, from an evolutionary perspective it may be advantageous for the virus to induce some IFN, at least in some cell types. This is because, once infection has been established, IFN is not able to eradicate the infection and instead may serve to drive chronic immune activation detrimental to the host. In T cells on the other hand, the inability to secrete IFN in response to HIV, and DNA in general, and mount antiviral responses may be important in allowing HIV to establish highly productive infection with massive virus production and spread throughout lymphoid tissues and the circulation.

In this study we have utilized a relatively homogeneous cell population consisting of activated T cells almost depleted of cells of the myeloid cell lineage. This imposes some limitations when drawing conclusions concerning the interplay between different cell types, which take place in lymphoid tissue and the circulation during HIV infection in vivo. Even though activated T cells do not produce IFN during HIV infection as suggested from this and a number of other studies, other sources of IFN may be present in lymphoid tissue or mucosa. Such type I IFN would particularly be derived from pDCs, and would serve to limit HIV replication within both myeloid and lymphoid cell lineages [11], [69]. In agreement with this idea, we found that adding exogenous type I IFN did inhibit HIV replication in activated T cells, supporting the idea that a functional DNA signaling machinery in T cells would endow this cell type with an extra means of cell-autonomous anti-HIV activity. Therefore, the final outcome of IFN-mediated effects upon viral replication may be dependent on the cellular context and whether IFN-producing cells are present in the vicinity of productively infected T cells.

In conclusion, we here demonstrate that the presence of cytosolic DNA in T cells activates upstream events in the innate DNA sensing machinery ssDNA including recruitment of the DNA receptor IFI16 and assembly of foci containing STING and TBK1. However in contrast to what is observed in macrophages, DNA sensing does not lead to induction of innate responses in activated T cells, including expression of type I IFNs, ISGs or pro-inflammatory cytokines. Accordingly, we demonstrate that DNA transfection does not induce antiviral activity sufficient to inhibit HIV replication in infected T cells. Further studies are needed to clarify at which specific point DNA signaling is disrupted in T cells and also to establish whether this phenomenon plays a role in the permissiveness of activated CD4+ T cells for HIV.

An overall question raised by the present study is why T cells have a defect DNA signaling machinery, which is functional in most other cell types studied. It has been proposed that genomic replication generates DNA by-products with the ability to stimulate innate immune responses. Hence, it is tempting to speculate that T cells, which proliferate and perform DNA rearrangement of the T cell receptor after antigen-stimulation, need to have a dampened response to DNA in order to avoid development of DNA-driven inflammatory diseases.

## Materials and Methods

### Ethics statement

The work contains human studies. Approval was received from the local ethical committee (Committee for Research Ethics for Mid-Jutland County, permission number M-20110108) and informed written consent from all participating subjects was obtained.

### Cells

Human PBMCs were isolated from blood samples from healthy donors using Ficoll-paque (GE Healthcare). The blood was placed on top of a layer of Ficoll-paque and centrifuged at 1000×g for 20 min. Cells from the interphase layer were harvested, and washed twice in PBS, and re-suspended in complete RPMI (RPMI-1640 medium containing 10% heat inactivated FCS, l-glutamine, and streptomycin and penicillin). For activation, the PBMCs were pre-incubated in a culture flask at a density of 2×10^6^ cells/mL for 48 hours in complete RPMI supplemented with PHA (5 µg/mL) and interleukin (IL) 2 (20 U/mL). For the following 24 hours of pre-incubation, and during experiments, cells were kept in complete RPMI supplemented in IL2. For experiments, PBMCs were seeded in 24 well plates at a density of 1×10^6^ cells per well. THP-1 cells were grown in complete RPMI in culture flasks. For experiments, cells were seeded in 24 well plates at a density of 400.000 cells per well in the presence of 100 nM PMA for differentiation. 18 hours prior to experiments. CD4+ T cells were purified from PBMCs using EasySep® Human CD4+ T Cell Enrichment Kit (StemCell Technologies) with a purity of 98%, and grown in complete RPMI. For generation of monocyte-derived macrophages (MDM)s buffy coats from Aarhus University Hospital blood bank were used to collect PBMCs by Ficoll Paque (GE Healthcare) gradient centrifugation. Monocytes were then separated from PBMCs by plastic adherence in RPMI 1640 supplemented with 10% (v/v) AB-positive human serum (Invitrogen), 15 ng/ml M-CSF (Sigma), 600 µg/ml Glutamine, 200 IU/ml penicillin and 100 µg/ml streptomycin (Gibco). Monocytes were allowed to differentiate into MDMs for 7 days.

### Virus

HIV-1 BaL was generated from a pWT/BaL plasmid (NIH AIDS research and reference reagent program cat.no 11414), containing a full-length HIV-1 provirus, consisting of the backbone of the HXB-3 strain of HIV-I iiiB containing 2687bp Sal I to Bam HI fragment from strain BaL, including BaL Tat, Rev, Vpu and gp120 sequences plus part of Vpr and gp41. The plasmids were amplified in One Shot Stbl3 cells (Invitrogen) and purified with Qiagen Plasmid Plus Kit. Plasmids were transfected into 293T cells using Fugene-6 (Roche) as described by the manufacturer. Supernatants were harvested after 48 and 72 hours, filtrated through a 0.4 µm filter and ultra-centrifuged at 100.000×g over a 25% sucrose cushion. The viral pellet was re-suspended in RPMI, and the titer was determined by a luciferase based assay using TZM-bl cells. Briefly, the virus was titrated onto TZM-bl cells with 8 replica of each viral concentration in a 96 well plate. After 4 days of infection, cells were lysed using 90 µL 0.5% NP40 in PBS per well and incubated for 45 min. 90 µL Britelite Plus Reagent (PerkinElmer) was added to each well, and the plate was red immediately in a luminometer. The TCID50 was calculated using Reed Munch calculation. In parallel with HIV-1 pWT/BaL production a control plasmid pUC19 was also transfected into 293T cells, subjected to the same procedures as the HIV-1 strain and used in experiments as a control for potential contaminants derived from the 293T cells, from the pUC19 plasmid or plasmid preparation. Sendai virus, strain Cantell (kindly provided by Ilkka Julkunen, Helsinki) was grown in 11-day-old embryonated hen eggs, as previously described [70]. The infectivity titer of the virus in DCs was 4×10^9^ PFU/mL. The uninfected hen egg allantoic fluid did not stimulate pro-inflammatory cytokine expression in DCs, and the virus preparation did not contain lipopolysaccharide.

### HIV infections

Cells were seeded in 24-well plates at a density of 1×10^6^ cells per well. Virus was added at a concentration of 0.002 MOI, reaching a total volume of 400 µL. To maximize infection, cells were left incubating 1 hour at 37°C, followed by centrifugation at 1200×g at 4°C for 1 hour. After 2 hours of incubation, cells were washed twice in pre-warmed RPMI to remove non-fused virus, re-suspended in 700 µL complete RPMI and incubated for various time periods ranging from 6 hours-72 hours.

### DNA oligos

For DNA transfections, we used a ssDNA derived from HIV [53], and a dsDNA sequence derived from HSV-1 [33] (both from DNA Technology). The HIV-derived DNA used was derived from the 5′-UTR: ssDNA, 100 bases: 5′ GTC TCT CTG GTT AGA CCA GAT CTG AGG CAG CCT CAG ATG GCT AAC TAG GGA GAC CAC TGC TTA AGC CTC AAT ACA GCT TGT ATT GAG GCT TCA AGT AGT G. The DNA derived from HSV-1 was dsDNA, 60 bases forward: 5′ TAA GAC ACG ATG CGA TAA AAT CTG TTT GTA AAA TTT ATT AAG GGT ACA AAT TGC CCT AGC, and dsDNA reverse: GCT AGG GCA ATT TGT ACC CTT AAT AAA TTT TAC AAA CAG ATT TTA TCG CAT CGT GTC TTA. DNAs were annealed in annealing buffer containing 100 mM KCl, 20 mM HEPES pH 7.4, by mixing the complementary DNA strands, or the ssDNAs alone, and heating them at 95°C for 5 min, followed by a slow cool-down at room temperature for 1.5 hours. DNA was kept at 4°C until use in experiments. The TLR9 agonist ODN2216 was obtained from InVivoGen.

### Reagents

Stimuli for cell activation, and control stimuli in experiments, were added directly into the medium or transfected into the cells using the same procedure as for the DNA oligos. IL2 (20 U/mL), IFNβ (1 ng/mL and 10 ng/mL), were obtained from Invitrogen. PHA (5 µg/mL) was obtained from Gibco, and PMA (100 nM) from Invivogen. The antiretroviral drug azidothymidine (AZT), obtained from the NIH AIDS Reagent Program, was used at a concentration of 0.2 µM, 2 µM, and 20 µM. These concentrations correspond to 1, 10, and 100×IC50 [71].

### DNA transfections

DNA oligos were transfected into the cells using lipofectamine 2000 (Invitrogen). The DNA and lipofectamine were mixed at a ratio of 1 µg DNA per 1 µL lipofectamine. DNA and lipofectamine were mixed in OptiMEM (Invitrogen) and allowed to form complexes for 20 min. The mixture was added to cells reaching a final DNA concentration of 2 µg/mL. ODN2216 was added directly to the culture medium without the use of lipofectamine.

### RNA analysis

RNA was collected using High Pure RNA Isolation kit (Roche). cDNA synthesis was performed using M-MLV RT (Invitrogen). Inductions of ISG56, CXCL10 and GAPDH were measured with QuantiFast SYBR Green Kit (Qiagen) and the following primer sequences were used: ISG56 forward: CCT CCT TGG GTT CGT CTA CA/reverse: GGC TGA TAT CTG GGT GCC TA; CXCL10 forward: AGG AAC CTC CAG TCT CAG CAC CA/reverse: TGC TGA TGC AGG TAC AGC GTA CA; GAPDH forward: TCT TTT GCG TCG CCA GCC GAG/reverse: ACC AGG CGC CCA ATA CGA CCA. IFNβ and β-actin were measured using a premade Taqman assay and an RNA to Ct one step kit (Applied Biosystems). Taqman probe was Hs01077958_s1 from Life Technologies. The target genes were normalized to either GAPDH or β-actin expression as housekeeping genes, and to the relevant control.

### ELISA

Viral replication was measured by an in-house p24 ELISA using affinity-purified anti-HIV-1-p24 (Aalto D7320) and biotinylated conjugate of anti-HIV-1-p24 mAb (Aalto BC1071-BIOT). CXCL10 was measured using a human CXCL10/IP-10 ELISA kit (R&D systems), according to their manual.

### Luminex

The supernatants from PBMCs pretreated with IL2/PHA stimulated with dsDNA, ssDNA, ssDNA2 (2 µg/mL) and Sendai virus incubated 24 hours were evaluated for following cytokines. IFNα2, IL6, MIPα, CXCL10 and TNFα levels were measured using a human Bio-Plex Cytokine multiplex assay (Bio-Rad), following the manufacturer's instructions. For measurements of intracellular p-IκBα levels, cells were lysed after 6 hours of stimulation using Bio-Plex cell Lysis kit (Bio-Rad), and p-IκBα levels were measured using p-IκBα Bio-Plex Phospho-protein Assay (Bio-Rad).

### Confocal microscopy

PBMCs were seated on Poly-L-lysine coated coverslips in a 48 well plate at a concentration of 9×10^5^ cells/ml and then transfected with DNA conjugated directly to fluorescein isothiocyanate (FITC). After 1 or 4 hours of incubation, the cells were fixed in 4°C methanol for 5 min in −20°C and treated 1 min with 0,01% Triton X-100 to permeabilize the cells. Coverslips were pre-incubated in 1% BSA in PBS, and stained for 1–2 hours at 25°C with primary antibodies (1∶25–100 dilution) and for 1 hour with secondary antibodies (1∶500 dilution). Primary antibodies used: anti-IFI16 (1G7; Santa Cruz), anti-CD3 (MCA1477; AbD), anti-STING (IMG-6422A; Imgenex) or (MAB7196, R&D Systems), anti-TBK1 (L-15, Santa Cruz) Alexa Flour -405, 568, 647 and Dylight-405 labeled secondary antibodies. Coverslips were mounted in Prolong Gold (Invitrogen). Images processing was performed using Zen 2012 (Zeiss) and ImageJ.

### Western Blotting

PBMCs pretreated with IL2/PHA were lysed with 200–400 µl 2× Laemmli buffer and 10 mM DTT. 10 µl of each cell lysate was run on a Bio-Rad premade Criterion gel (Ready Gel 10% Bis-Tris Gel, Bio-Rad) and transferred onto PVDF membranes. Protein levels were determined with the following specific anti-bodies: IFI16 (1G7, Santa Cruz); cGAS (HPA031700, Sigma); STING (MAB7196, R&D Systems); TBK1 (L-15, Santa Cruz); IRF3 (FL-425, Santa Cruz); phospho-TBK1 (D52C2, Cell Signaling), and β-actin (AC-15 HRP, Abcam).

### Flow cytometry

Three different antibody panels were used to analyze surface expression markers and activation markers of PBMCs: Panel A: anti-CD3 FITC, anti-CD25 PE, anti-CD4 APC, anti-CD38 PerCP-Cy5.5, anti-HLA-DR PE-Cy7, and a viability marker (Live Dead near IR). Panel B: anti-CD3 FITC, anti-CD4 PerCP-Cy5.5, anti-CD25 PE, anti-CD69 APC, and a viability marker (Live Dead near IR). Panel C: anti-CD3 FITC, anti-CD14 APC, anti-CD4 PE, anti-CD19 PerCP, and a viability marker (Live Dead near IR). Antibodies were all purchased from Biolegend and BD Biosciences. Cells were fixated and permeabilized for intracellular staining using BD Fixation/Permabilization kit according to the manufacturer's instructions. Data were collected on a FACSCanto flow cytometer (BD Biosciences). Samples were compensated for spectral overlap, using BD CompBeads (BD Biosciences) and relevant antibodies.

### Caspase assay

Caspase activity was measured using a flow-based multi-caspase FAM kit from Guava Technologies (Cat. No 4500-0530). It is designed to identify apoptotic cells and to discriminate between mid- and late apoptotic cells. The kit contains an inhibitor specific for the caspase 3, 7, 8, and 9 conjungated to FAM flurochrome. This reagent binds to activated caspases and remains inside the cell, while unbound reagent is washed away during cell preparation. The resulting fluorescence signal is proportional to the number of activated caspase enzymes present in the cell. 7-AAD was used as viability marker.

### Statistical analysis

Data was analyzed and plotted using FlowJo (version 9.5.2, Tree Star, Ashland, OR), and GraphPad Prism 5.0 for Mac OS X, (GraphPad Software, San Diego, CA). Data presented are means +/− standard deviations (SD).

## Supporting Information

Figure S1 **IL2/PHA PBMCs mainly consist of highly activated T cells.** Human PBMCs freshly isolated and stimulated with PHA (5 μg/mL) and IL2 (20 U/mL) for 48 hours followed by 24 hours of IL2 stimulation were stained with specific antibodies for CD3, CD4, CD14, and CD19, and analyzed by flow cytometry. (**A**) A forward- versus side-scatter and subsequent forward-scatter area versus height was used to define events representing single cells. Dead cells were excluded from further analysis in a forward-scatter versus Live-Dead near IR (detected in the APC-Cy7 detector). (**B**) Histogram representing the distribution of cell surface expression based on the flow cytometric analysis. Plot represents data from two independent experiments and is presented as mean +/− SD. (**C**) PBMCs were and either stimulated with IL2/PHA as described above, or left un-stimulated for the same amount of time. After 3 days, the cells were stained for CD3 and the following activation markers: HLA-DR, CD38, CD25, and CD69, and analyzed by flow cytometry. Plots represent activation markers on CD3+ cells in one experiment. Similar results were obtained in two independent experiments.
(PDF)

Figure S2 **Transfection efficiency of IL2/PHA PBMCs.** IL2/PHA PBMCs were mock transfected or transfected with FITC labelled ssDNA. After 1 hour of incubation, cells were fixed, stained, and analysed. A forward- versus side-scatter and subsequent forward-scatter area versus height was used to define events representing single cells. Dead cells were excluded from further analysis in a forward-scatter versus Live-Dead near IR (detected in the APC-Cy7 detector). Plot represents FITC expressing CD3+ cells from one donor. Similar results were obtained in 4 independent experiments.
(PDF)

Figure S3 **IL2/PHA PBMCs express IFN-stimulated genes after stimulation with the TLR9 agonist ODN2216.** IL2/PHA PBMCs were treated with ODN2216 (3 µM) or infected with SeV (MOI 0.5). Supernatants were harvested after 24 hours and analyzed for CXCL10 protein levels. Data are shown as means of triplicates +/− SD. Mock, Lipofectamine. Similar results were obtained with two independent donors.
(PDF)

Figure S4 **Viability and activation markers on CD4+ T cells stimulated by IL2/PHA.** CD4+ cells isolated from PBMCs were left un-stimulated (**A–E**) or stimulated with IL2/PHA (**F–J**) and characterized by flow cytometry. For un-stimulated CD4+ cells: (**A**) forward-side scatter plot and (**B**) 7-AAD versus side scatter plot to detect live versus dead cells. (**C–E**) The cells were stained with specific antibodies for CD4, CD25, and CD69, and analyzed by flow cytometry. For CD4+ cells stimulated with IL-2/PHA: (**F**) forward-side scatter plot and (**G**) 7-AAD versus side scatter plot to detect live versus dead cells. (**H–J**) The cells were stained with specific antibodies for CD4, CD25, and CD69, and analyzed by flow cytometry. Plots represent data from one donor. Similar results were obtained in two independent experiments.
(PDF)

Figure S5 **Presence of cytoplasmic DNA does not induce pro-apoptotic pathways in IL2/PHA PBMCs.** IL2/PHA PBMCs were transfected with ssDNA (2 µg/mL) or treated with Etoposide (20 µM) for 24 hours. Cells were analysed using a flow cytometry-based multi-caspase kit detecting activity of caspase 3, 7, 8, and 9, as well as dead cells. Data plotted represent one experiment. Similar results were observed using samples isolated after 4 hours of stimulation.
(PDF)

Figure S6 **Confocal microscopy images of IL2/PHA PBMCs.** IL2/PHA PBMCs were (**A**) fixed and stained with anti-CD3 antibody or (**B**) transfected with 2 µg/mL of FAM-labeled HIV-Tar RNA for 2 hours, then fixed and stained with anti-IFI16 antibody.
(PDF)
