# Supplementary figures and images for: T Cells Detect Intracellular DNA but Fail to Induce Type I IFN Responses: Implications for Restriction of HIV Replication
Source: PLoS One. 2014 Jan 3;9(1):e84513. doi: 10.1371/journal.pone.0084513 (PMC3880311; doi:10.1371/journal.pone.0084513)

**A**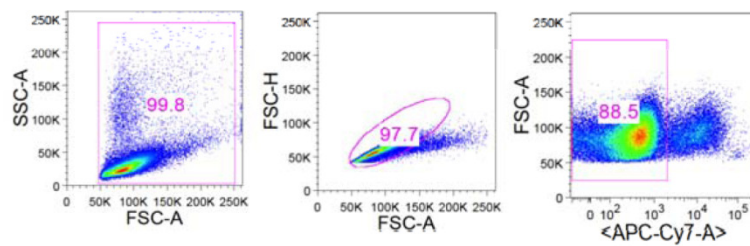**B**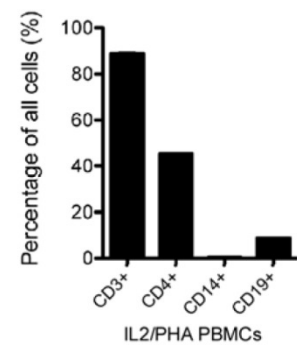**C**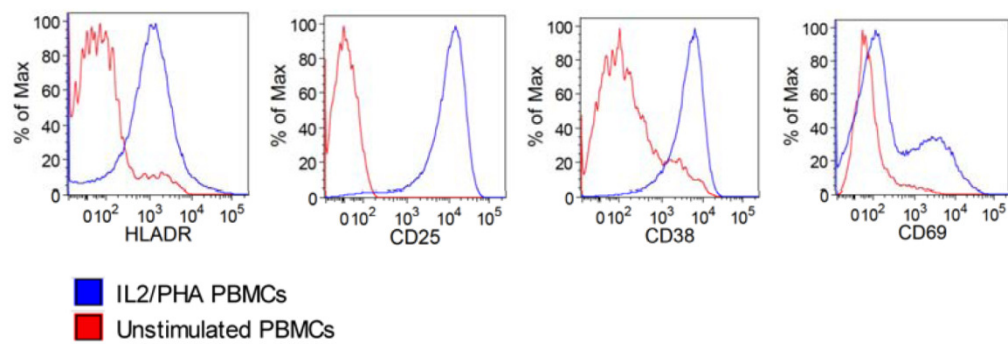

Supplement: Figure S1 — IL2/PHA PBMCs mainly consist of highly activated T cells. Human PBMCs freshly isolated and stimulated with PHA (5 μg/mL) and IL2 (20 U/mL) for 48 hours followed by 24 hours of IL2 stimulation were stained with specific antibodies for CD3, CD4, CD14, and CD19, and analyzed by flow cytometry. (A) A forward- versus side-scatter and subsequent forward-scatter area versus height was used to define events representing single cells. Dead cells were excluded from further analysis in a forward-scatter versus Live-Dead near IR (detected in the APC-Cy7 detector). (B) Histogram representing the distribution of cell surface expression based on the flow cytometric analysis. Plot represents data from two independent experiments and is presented as mean +/− SD. (C) PBMCs were and either stimulated with IL2/PHA as described above, or left un-stimulated for the same amount of time. After 3 days, the cells were stained for CD3 and the following activation markers: HLA-DR, CD38, CD25, and CD69, and analyzed by flow cytometry. Plots represent activation markers on CD3+ cells in one experiment. Similar results were obtained in two independent experiments. (PDF) [file pone.0084513.s001.pdf]

## MOCK

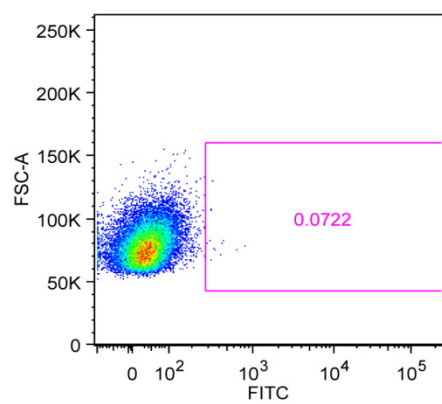

## ssDNA

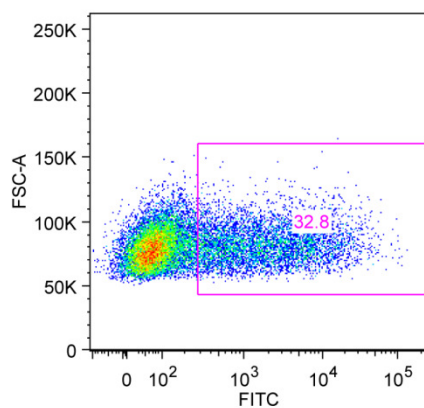

Supplement: Figure S2 — Transfection efficiency of IL2/PHA PBMCs. IL2/PHA PBMCs were mock transfected or transfected with FITC labelled ssDNA. After 1 hour of incubation, cells were fixed, stained, and analysed. A forward- versus side-scatter and subsequent forward-scatter area versus height was used to define events representing single cells. Dead cells were excluded from further analysis in a forward-scatter versus Live-Dead near IR (detected in the APC-Cy7 detector). Plot represents FITC expressing CD3+ cells from one donor. Similar results were obtained in 4 independent experiments. (PDF) [file pone.0084513.s002.pdf]

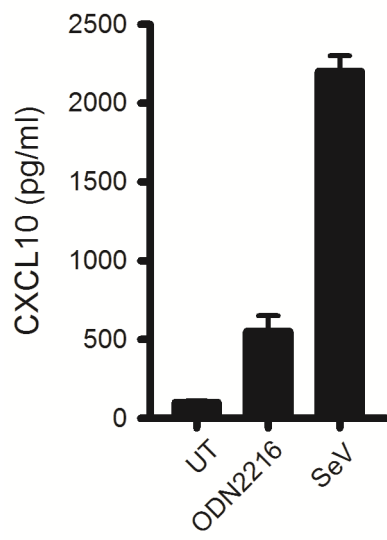

Supplement: Figure S3 — IL2/PHA PBMCs express IFN-stimulated genes after stimulation with the TLR9 agonist ODN2216. IL2/PHA PBMCs were treated with ODN2216 (3 µM) or infected with SeV (MOI 0.5). Supernatants were harvested after 24 hours and analyzed for CXCL10 protein levels. Data are shown as means of triplicates +/− SD. Mock, Lipofectamine. Similar results were obtained with two independent donors. (PDF) [file pone.0084513.s003.pdf]

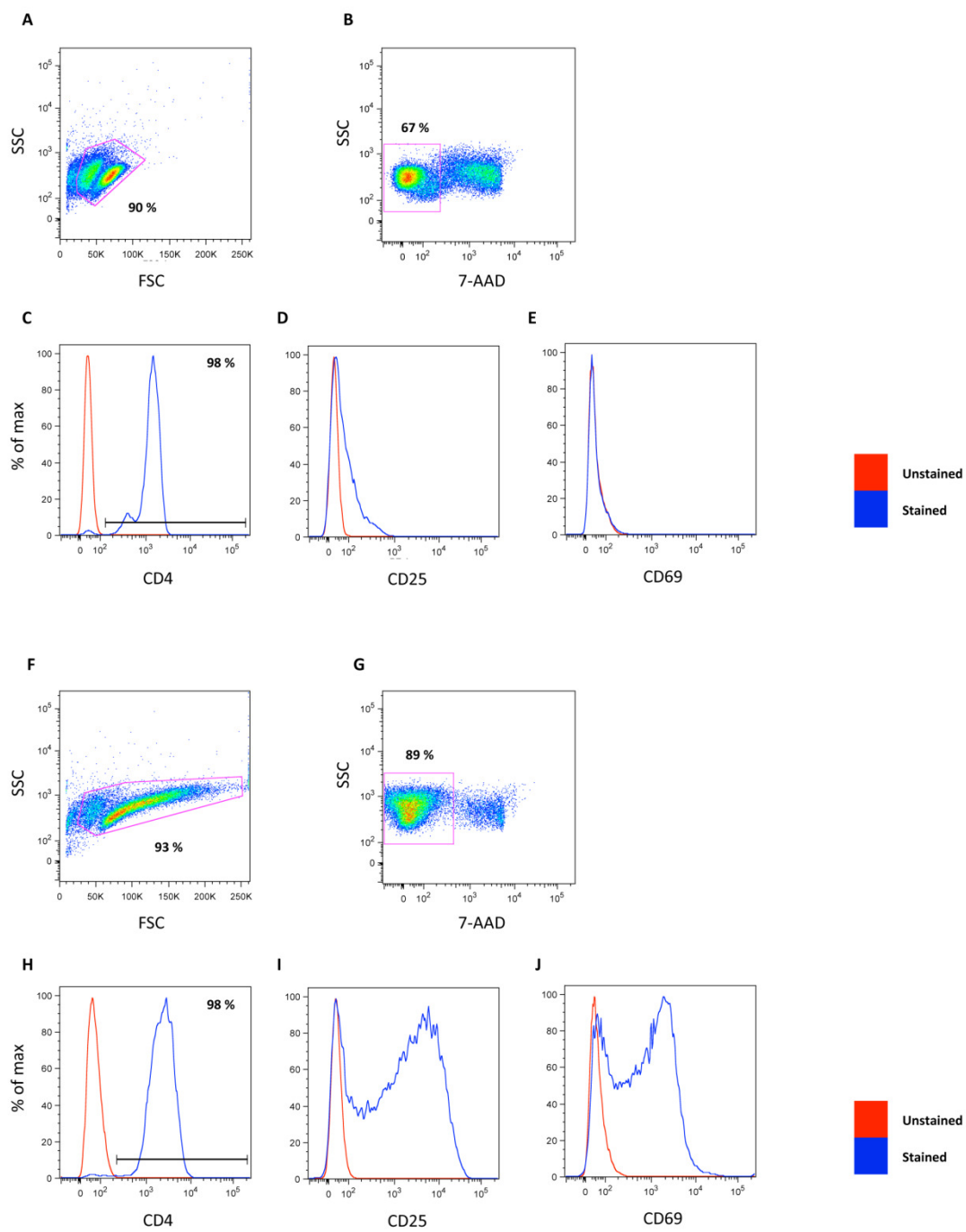

Supplement: Figure S4 — Viability and activation markers on CD4+ T cells stimulated by IL2/PHA. CD4+ cells isolated from PBMCs were left un-stimulated (A–E) or stimulated with IL2/PHA (F–J) and characterized by flow cytometry. For un-stimulated CD4+ cells: (A) forward-side scatter plot and (B) 7-AAD versus side scatter plot to detect live versus dead cells. (C–E) The cells were stained with specific antibodies for CD4, CD25, and CD69, and analyzed by flow cytometry. For CD4+ cells stimulated with IL-2/PHA: (F) forward-side scatter plot and (G) 7-AAD versus side scatter plot to detect live versus dead cells. (H–J) The cells were stained with specific antibodies for CD4, CD25, and CD69, and analyzed by flow cytometry. Plots represent data from one donor. Similar results were obtained in two independent experiments. (PDF) [file pone.0084513.s004.pdf]

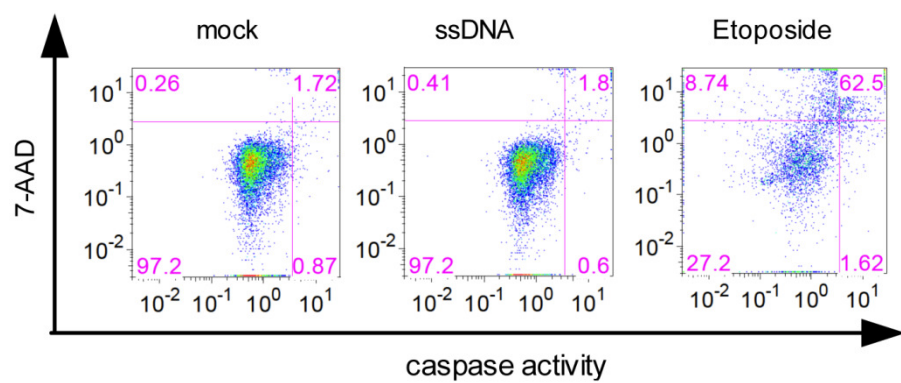

Supplement: Figure S5 — Presence of cytoplasmic DNA does not induce pro-apoptotic pathways in IL2/PHA PBMCs. IL2/PHA PBMCs were transfected with ssDNA (2 µg/mL) or treated with Etoposide (20 µM) for 24 hours. Cells were analysed using a flow cytometry-based multi-caspase kit detecting activity of caspase 3, 7, 8, and 9, as well as dead cells. Data plotted represent one experiment. Similar results were observed using samples isolated after 4 hours of stimulation. (PDF) [file pone.0084513.s005.pdf]

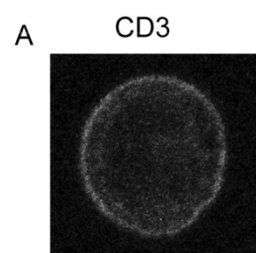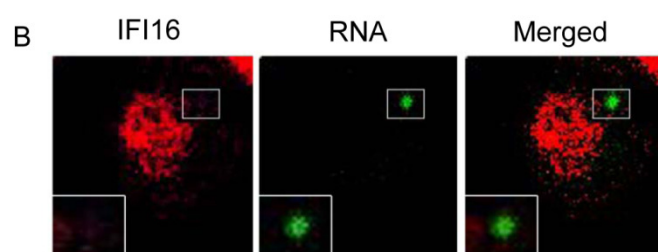

Supplement: Figure S6 — Confocal microscopy images of IL2/PHA PBMCs. IL2/PHA PBMCs were (A) fixed and stained with anti-CD3 antibody or (B) transfected with 2 µg/mL of FAM-labeled HIV-Tar RNA for 2 hours, then fixed and stained with anti-IFI16 antibody. (PDF) [file pone.0084513.s006.pdf]
